# Spectral organization of focal seizures within the thalamotemporal network

**DOI:** 10.1002/acn3.50880

**Published:** 2019-08-30

**Authors:** Diana Pizarro, Adeel Ilyas, Ganne Chaitanya, Emilia Toth, Auriana Irannejad, Andrew Romeo, Kristen O. Riley, Leonidas Iasemidis, Sandipan Pati

**Affiliations:** ^1^ Department of Neurology University of Alabama at Birmingham Birmingham Alabama; ^2^ Epilepsy and Cognitive Neurophysiology Laboratory University of Alabama at Birmingham Birmingham Alabama; ^3^ Department of Neurosurgery University of Alabama at Birmingham Birmingham Alabama; ^4^ Center for Biomedical Engineering and Rehabilitation Science Louisiana Tech University Ruston Louisiana

## Abstract

**Objective:**

To investigate dynamic changes in neural activity between the anterior nucleus of the thalamus (ANT) and the seizure onset zone (SOZ) in patients with drug‐resistant temporal lobe epilepsy (TLE) based on anatomic location, seizure subtype, and state of vigilance (SOV).

**Methods:**

Eleven patients undergoing stereoelectroencephalography for seizure localization were recruited prospectively for local field potential (LFP) recording directly from the ANT. The SOZ was identified using line length and epileptogenicity index. Changes in power spectral density (PSD) were compared between the two anatomic sites as seizures (*N* = 53) transitioned from interictal baseline to the posttermination stage.

**Results:**

At baseline, the thalamic LFPs were significantly lower and distinct from the SOZ with the presence of higher power in the fast ripple band (*P* < 0.001). Temporal changes in ictal power of neural activity within ANT mimic those of the SOZ, are increased significantly at seizure onset (*P* < 0.05), and are distinct for seizures that impaired awareness or that secondarily generalized (*P* < 0.05). The onset of seizure was preceded by a decrease in the mean power spectral density (PSD) in ANT and SOZ (*P* < 0.05). Neural activity correlated with different states of vigilance at seizure onset within the ANT but not in the SOZ (*P* = 0.005).

**Interpretation:**

The ANT can be recruited at the onset of mesial temporal lobe seizures, and the recruitment pattern differs with seizure subtypes. Furthermore, changes in neural dynamics precede seizure onset and are widespread to involve temporo‐thalamic regions, thereby providing an opportunity to intervene early with closed‐loop DBS.

## Introduction

Delineation of the epileptogenic zone using stereotactic electroencephalography (sEEG) relies on the accurate interpretation of the recorded electrophysiologic signals.^1^ Numerous contemporary signal processing techniques exist to facilitate this intricate interpretation; among these, the spectrographic analysis is a fundamental means of visualizing the temporal organization of energy in various frequency bands.^2^ Indeed, a salient feature of seizures is an abrupt redistribution of energy into higher frequency bands that is distinct from the interictal baseline, which can readily be detected by spectrographic analysis of local field potentials (LFP).^3^, ^4^ Such abrupt transitions also emerge from epilepsy models of coupled neuronal populations where decreased inhibition produces fast activity and ictal changes in LFP mimic in vivo sEEG recordings.^5^ Thus, analysis of LFPs in the time–frequency domain from recorded sEEG may serve well to delineate ictal activity at a neuronal population level within a region or a distributed network.^6^

Penfield and Jasper recognized the role of anatomically disparate subcortical structures including the thalamus, in ictogenesis.^7^, ^8^ The thalamic nuclei, through diverse reciprocal connectivity to the cortex, can regulate seizure initiation and termination. Several preclinical studies have demonstrated that the anterior nucleus of the thalamus (ANT) plays a role in the organization of seizure initiation and propagation and is targeted for open‐loop deep brain stimulation (DBS).^9^, ^10^, ^11^ Although power spectral changes in LFP recorded intracranially offers insight into ictal neural activity, the spatial sampling in patients has been confined to cortical and subcortical regions other than thalamus.^12^ To date, no clinical study has investigated peri‐ictal spectrographic changes in the thalamus in focal epilepsy.

There is a dearth of data regarding the spectral changes within the human thalamus during seizures. The neuronal architecture of the thalamus differs from that of cortical structures, and these differences have electrophysiologic ramifications.^13^, ^14^, ^15^ For example, the thalamus contains a diverse set of relay cells that express different firing patterns based on cortical inputs and, therefore, have a predisposition to organized spectral activity.^15^ In this study, we compare energy distribution between the epileptogenic cortex and the ANT during ictogenesis, focusing on differences in power spectral density (PSD) within these anatomically disparate regions for different seizure stages and types. We also compare the PSD for seizures that have onset during sleep versus wakefulness. Given differences in the cellular and functional organization of the mesial temporal regions and ANT, we anticipate they will manifest distinct PSD signatures during ictogenesis.

## Patients and Methods

### Patient Selection, consents, and protocol approval

**Figure 1:**
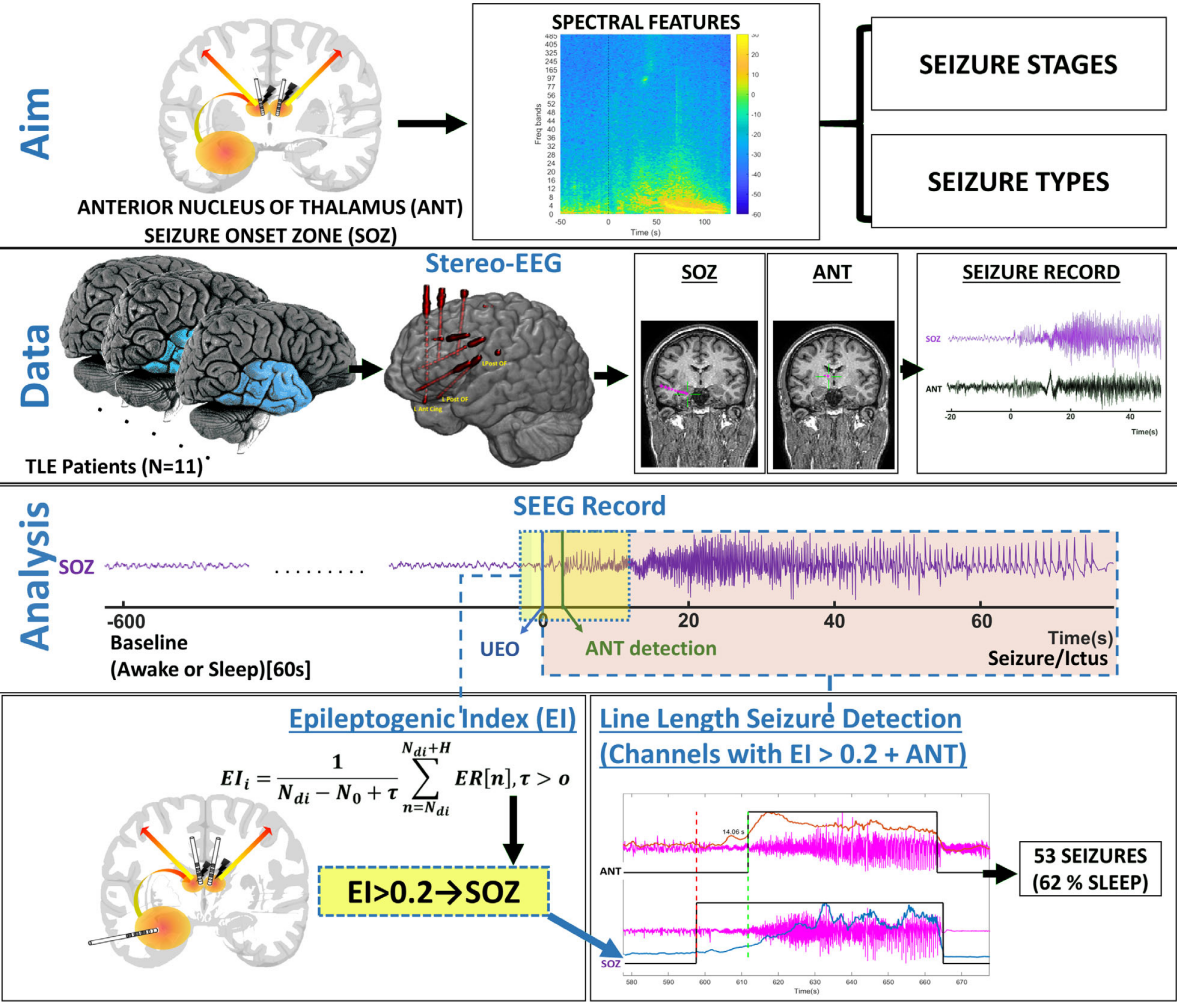
Schematic overview of the study with EEG data‐processing pipeline

The Institutional Review Board of the University of Alabama at Birmingham approved this study (IRB‐170323005). Each patient provided written consent for ANT electrode implantation and recording for research purposes. Patients with suspected temporal lobe epilepsy (TLE) underwent a presurgical evaluation that included a review of a detailed history, physical examination, neuropsychological assessment, video‐EEG, 3‐Tesla cerebral magnetic resonance imaging (MRI), magnetoencephalography, and interictal 18F‐fluorodeoxyglucose positron emission tomography.^16^ The decision to offer sEEG was based on discordant data between noninvasive studies (imaging, semiology, or electrophysiology) and the targets for sEEG were discussed in a multidisciplinary patient management conference. Beginning May 2017, all patients with suspected TLE undergoing sEEG implantation for seizure localization were eligible to participate in the study. The connectivity of ANT to the mesial limbic network and its potential ability to modulate limbic seizures influenced the decision to limit the study cohort to patients with TLE only.^9^, ^17^ The pipeline for data analysis is summarized in Figure 1.

### Recording of sEEG

**Figure 2:**
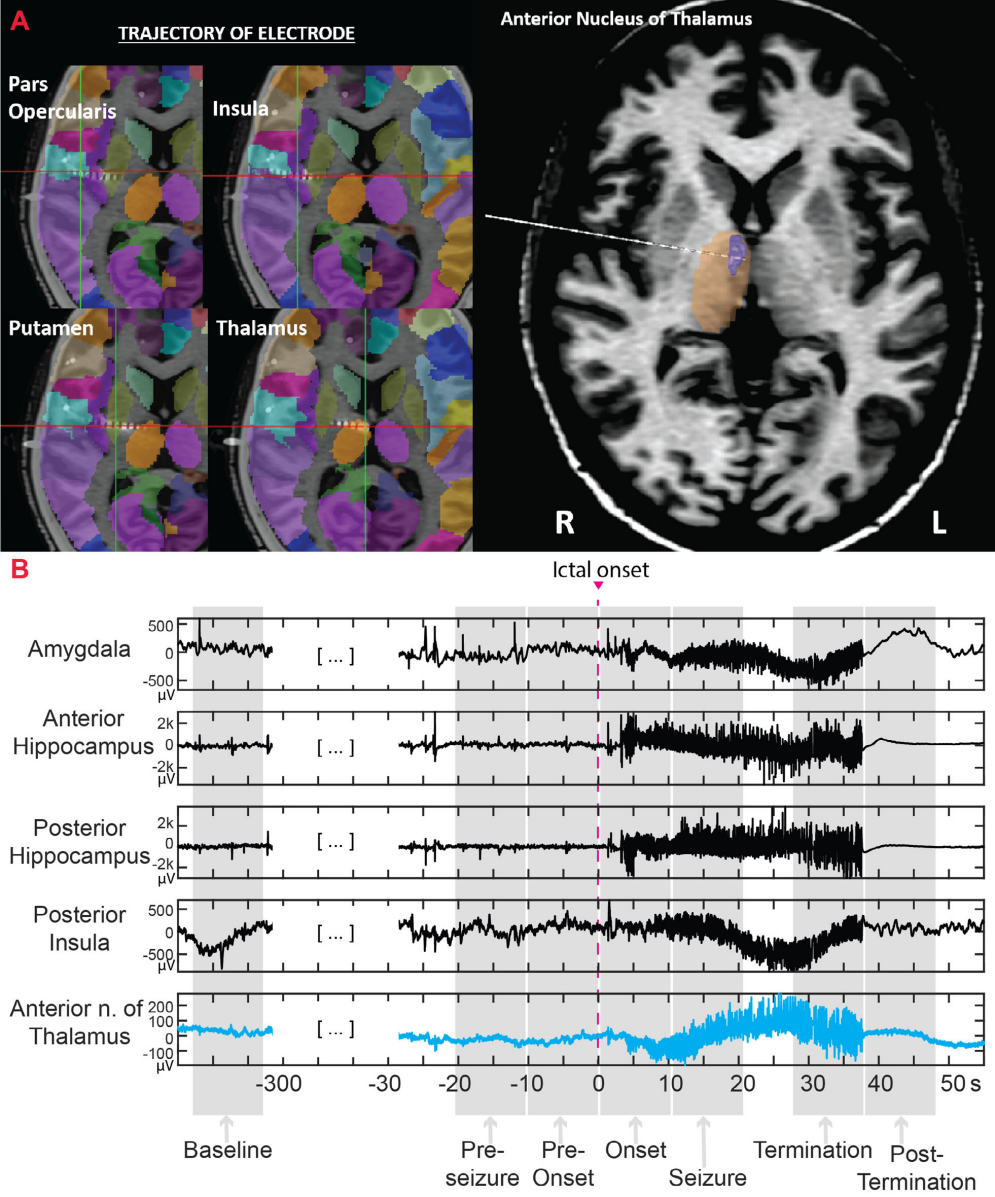
(A) This figure shows the implant strategy for implantation. The trajectory of one of the depth electrodes that was planned to target the opercular regions and insula (for clinical purpose) was modified to include sampling from the anterior nucleus of the thalamus (ANT)^19^, ^20^. (B) stereo‐EEG stages of the seizure with selected recordings from the anterior and posterior hippocampus, amygdala, posterior insula, and anterior thalamus. The ictal onset was at time 0 (highlighted with a vertical red line)

Electrodes consisted of cylindrical tubing (0.8 mm outer diameter) with 12–16 contacts per electrode, each of 2 mm length and 1.5 mm intercontact distance (PMT^®^ Corporation, Chanhassen, MN). Using robotic assistance (ROSA^®^ device, Medtech, Syracuse, NY), sEEG electrodes were implanted into predetermined regions of interest for seizure localization. The trajectory of one of the depth electrodes that was planned to target insula and operculum regions (for clinical purposes) was modified to include sampling from the ANT (Fig. 2). In this way, we avoided implanting additional depth electrodes for research purposes. Seizures from insular and operculum regions can mimic the presentation of temporal lobe seizures (“pseudo TLE”) or can be within the ictal onset network of mesial TLE (TLE plus syndrome). Hence, sEEG exploration in patients with suspected TLE often includes sampling of the insular‐operculum regions. The locations of the research and clinical contacts in all included subjects were confirmed by coregistering the postimplantation computed tomography (CT) imaging with the preimplantation T1‐weighted MRI using affine transformation generated by FMRIB’s linear registration tool FLIRT (FMRIB, Oxford, UK)^18^ For representation of the electrodes, we combined registration strategies in LeadDBS and iElectrodes to map the electrode trajectory and the final thalamic target. The cortical regions implanted were confirmed by coregistering the preimplant MRI and postimplant CT with AAL atlas and the thalamic subnuclei were identified using the Morel’s atlas (Fig. 2A).^19^, ^20^, ^21^

### Selection of Peri‐Ictal sEEG for spectral analysis

Video‐EEG was sampled at 2048Hz using Natus Quantum (Natus Medical Incorporated, Pleasanton, CA). Seizures were annotated clinically by a board‐certified epileptologist (SP) and were categorized into different subtypes: electrographic (ES), focal onset aware (FAS), focal onset with impaired awareness (FIAS), focal to bilateral tonic‐clonic (FBTCS). Electrographic seizures were defined as the emergence of rhythmic or repetitive epileptiform spikes lasting more than 10s without clinical manifestations, and seizure onset was defined as the earliest occurrence of rhythmic or repetitive epileptiform spikes that preceded the behavioral onset.

Since the study was focused on comparing peri‐ictal spectral changes between ANT and SOZ, we analyzed only seizures with confirmed recruitment of ANT using the measure of line length (LL)^22^, ^23^ and visual inspection. The Line Length feature is a simplification of the Katz’s running fractal of a signal and is proportional to changes in amplitude and frequency variations of a seizure. The specific parameters used in this study were a moving average window of 0.25 sec with a 50% overlap. The threshold of detection was set at two standard deviations above the mean LL of a 4‐minute baseline segment, and the algorithm flagged a segment if the LL was persistently above the threshold for at least 10 sec. For each seizure, the seizure onset zone (SOZ) was identified quantitatively and visually. First, the epileptogenicity index (EI) was computed across all channels during seizure onset as previously described.^6^ The EI statistically summarizes the spectral and temporal parameters of sEEG signals and relates to the propensity of a brain area to generate low voltage fast discharges. Based on EI value (EI> 0.2), a subset of highly epileptogenic channels was selected for LL‐based seizure detection analysis.^24^ Within this subset, the anatomic region corresponding to the channel with the earliest LL‐based seizure onset detection was defined as the SOZ. Second, visual inspection was performed to confirm unequivocal electrographic changes at seizure onset within the identified SOZ channel. Ictal recruitment of ANT was determined when the LL became higher than the mean + 2SD of a 4‐minute baseline segment. Third, each seizure was classified based on the patient’s state of vigilance (SOV) before the ictal onset. Two to five seizures with confirmed ANT recruitment were randomly selected per subject for further analysis with the inclusion of at least one seizure of each subtype if the patient had multiple seizure subtypes.

Peri‐ictal sEEG segments from the SOZ and ANT channels were divided into five stages as follows:^25^ *pre‐seizure*, *pre‐onset*, *onset,* during *a seizure*, *termination*, and *post‐termination* (Fig. 2B). The *pre‐*seizure stage consisted of 10s of recording immediately before the *pre‐onset* stage. The *pre‐onset* stage began with the earliest EEG changes suggestive of seizure activity and terminated at the ictal onset and was the only stage of variable duration. The *onset* stage was defined as the first 10s of ictal activity, *seizure* the 10s following the *onset* stage, *termination* the 10s before seizure termination, and *post*‐*termination* the 10s following seizure termination. A *baseline* segment was also selected as a spike‐free 10s segment at least 60s before the pre‐onset stage (Fig. 2B).

### Estimation of power spectral density (PSD)

The sEEG recordings were referenced to the mastoid electrodes. A bipolar montage was used across consecutive contacts within each depth electrode. One bipolar channel was selected in SOZ and one in ANT for every seizure. The sEEG data were preprocessed using a 60‐Hz digital notch filter and a 2‐s detrending. For each sEEG stage, PSD was computed by Fast Fourier transform on 3‐s consecutive segments with 2‐s overlap (Welch’s method with Hamming window) per seizure and *baseline* stage from both SOZ and ANT channels. The PSD values were averaged per frequency band and then z‐normalized concerning the mean of the baseline segment to ensure comparability across stages. Segments that contained artifacts, such as motion, disconnection of electrodes, or 60 Hz harmonics, were removed from further analysis. The z‐normalized PSD was estimated in the following frequency bands: slow delta (0.1–1Hz), delta (1–4Hz), theta (4–8Hz), alpha (8–13Hz), beta (13–30Hz), gamma (30–70Hz), high gamma (70–120Hz), ripples (120–250Hz), and fast ripples (250–500Hz). In this manner, both SOZ and ANT channels in each frequency band, seizure stages, and seizure subtypes had an associated normalized mean PSD value.

### Statistical analysis

The raw PSD values were found to be not normally distributed (positively skewed and mesokurtic). Therefore, we log‐transformed (log10) the z‐normalized PSD values data. T‐tests with FDR correction (false discovery rate detection), and analysis of variance (ANOVA) with Bonferroni correction, were performed by taking the stage and frequency band‐specific PSD values as dependent variables and the following variables as independent: (1) anatomic location (SOZ vs. ANT), (2) seizure stage, (3) seizure type, and (4) level of vigilance (awake vs. sleep). T‐test with FDR correction was performed to identify the frequency‐specific significant changes between the thalamus and SOZ for each stage of seizure.

### Data availability

Due to patient privacy‐related issues, the IRB has not approved for the data to be available to the public. However the data can be available through a data sharing agreement and IRB approval between two partnering institutes and investigators.

## Results

### Seizure characteristics

The demographic and clinical characteristics of the participating patients are summarized in Table S1. Out of 71 seizures recorded in 11 subjects. 53 seizures (75%) were detected by LL within first 10 sec of seizure‐onset in the cortex. These 53 seizures (8 ES, 9 FAS, 21 FIAS, 15 FBTCS) were analyzed and reported in the study. Of these seizures, 33 (62%) arose from sleep. The regions identified as SOZ for this study were – anterior or posterior hippocampus, amygdala, anterior cingulate, temporal pole.

### Interictal PSD differences between thalamus and SOZ

**Figure 3:**
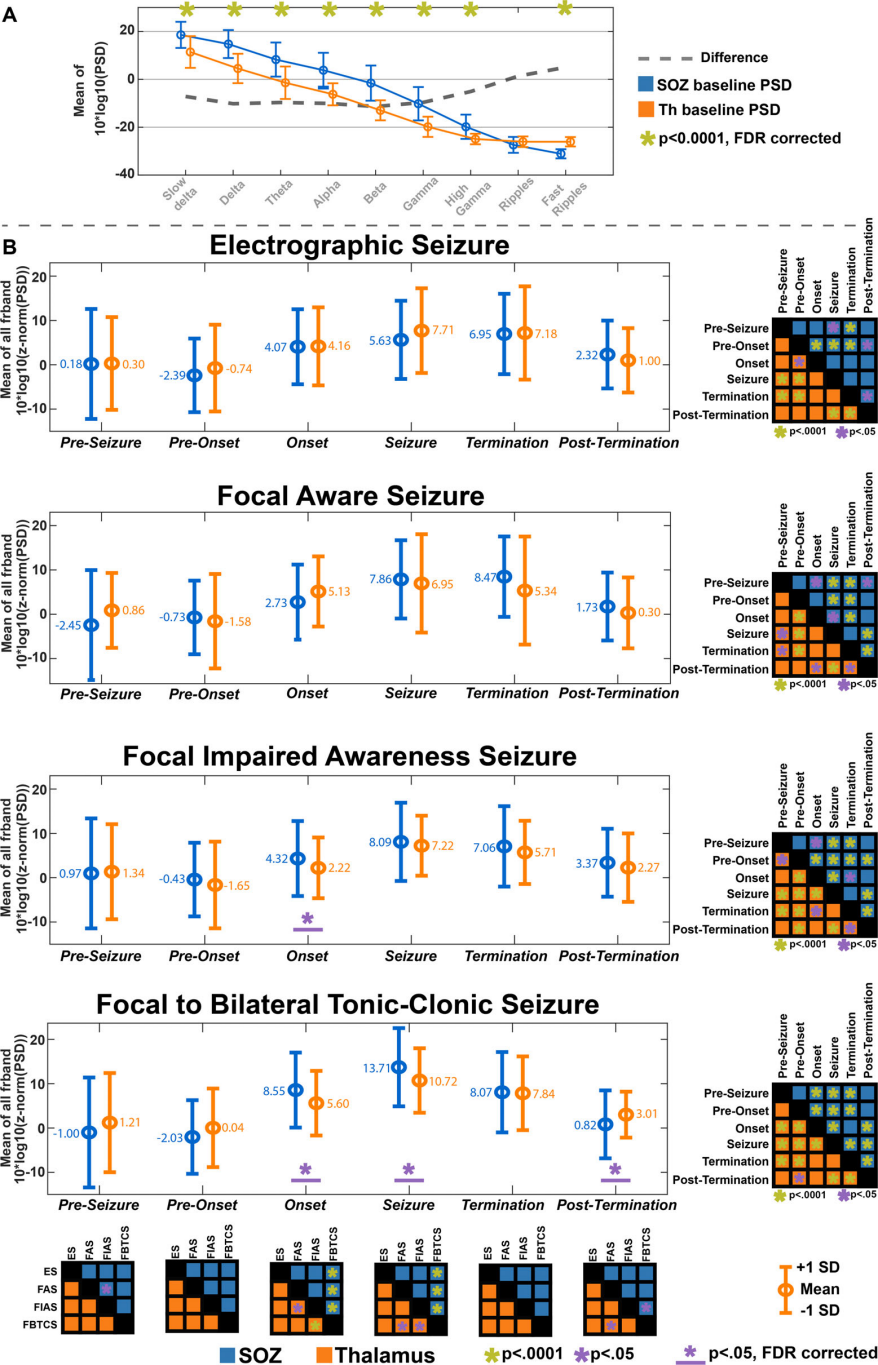
(A) Comparison in power spectral density (PSD) between seizure onset zone (SOZ) and anterior nucleus of the thalamus (ANT) at baseline. (B) Changes in PSD between SOZ and ANT for different seizure stages and seizure types. Comparison between seizure stages is provided in a matrix in the right corner for each seizure type. Comparison between seizure types for every seizure stage is provided in a matrix below

Examples of the thalamic and SOZ *baseline* PSDs are shown in Figure 3A. Compared to SOZ, the ANT manifested lower broadband (0.1–120 Hz) power (*t* < −6.08, *P* < 0.001), no significant difference in the ripple band (*t* = 2.42, *P* = 0.017), and higher PSD in the FR band (250–500Hz) (*t* = 13.29, *P* < 0.001).

### Peri‐ictal PSD differences between thalamus and SOZ

**Figure 4:**
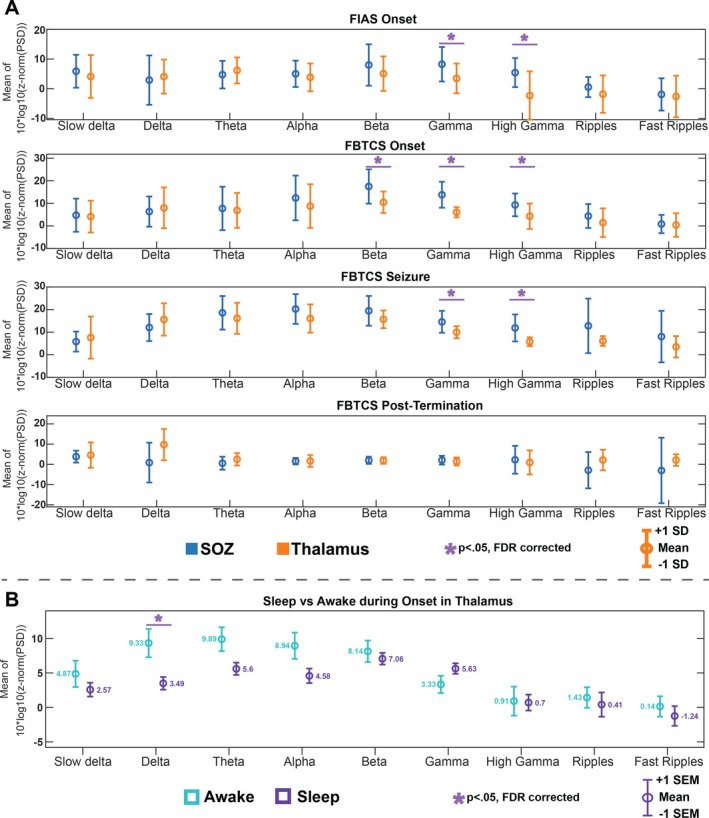
Frequency‐specific changes in power spectral density (PSD) between SOZ and ANT for seizure type and stages (A) Highlights the statistically significant differences between ANT and SOZ. Both focal onset with impaired awareness (FIAS) and focal to bilateral tonic‐clonic (FBTCS) at onset showed a lower PSD in ANT compared to SOZ. FBTCS continue to show this pattern through seizure and posttermination stages. This difference was noted to be driven by changes in gamma and high gamma frequencies, as well as beta for FBTCS during onset. (B) PSD of ANT was higher at seizure onset when awake in the delta frequency

The ANOVA results of the PSD comparison between ANT and SOZ for anatomic location, seizure type, and seizure stage are shown in Figure 3B. Spectral power began to rise during the *onset* phase, peaked during the *seizure* phase, and began to fall during the *post‐termination* in both ANT and SOZ. Although spectral changes were generally similar between ANT and SOZ, significant differences were noted in FIAS and FBTCS seizures at *seizure onset*, with the cortex having higher mean power than the thalamus (*t* > 3.07; pFDR < 0.002) in beta, gamma, and high gamma bands (Fig. 4A). In FBTCS seizures, there were differences between ANT and SOZ during the *seizure* (*t* = 2.94;pFDR = 0.004) in gamma and high gamma band, and *postictal* stages (delta bands; *t* = −2.76; pFDR = 0.006) (Fig. 4A). Interestingly, the *post‐ictal* stage in FBTCS had significantly higher mean power in ANT than SOZ (Fig. 4A).

### Peri‐ictal PSD changes within the thalamus

**Figure 5:**
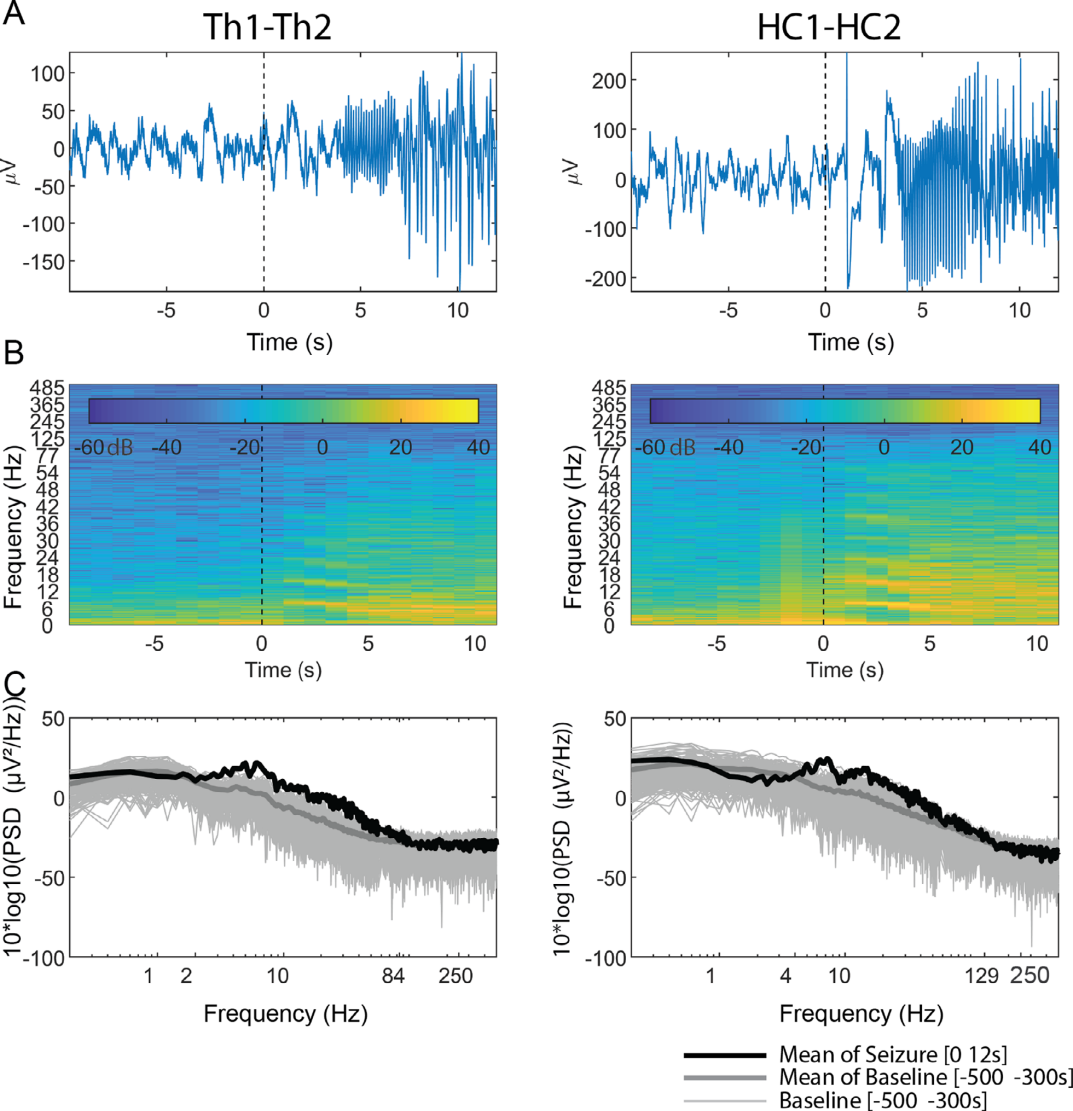
Changes in spectral power in SOZ and ANT at seizure onset. (A) stereo‐EEG recording from the hippocampus (HC) and ANT for first 10 seconds after seizure onset; (B) time–frequency decomposition of that sEEG signal; and (C) spectral power of that first 10 seconds after seizure onset. The baseline confidence represents a 95% confidence interval

The mean PSD in ANT was higher at the *onset* in comparison to the pre‐onset stages for all seizure types (*p‐Bonferroni* < 0.05) (Fig. 5). Interestingly, it was noted that the mean PSD in ANT during *pre‐onset* stage was the lowest compared to the *onset*, *seizure, and termination* windows (*P* < 0.05) (z‐normalized mean PSD < 0) (Fig. 4B). Midseizure recorded the highest increase in PSD of thalamus for all types of seizures.

### State of vigilance influences thalamic activity at seizure onset

**Figure 6:**
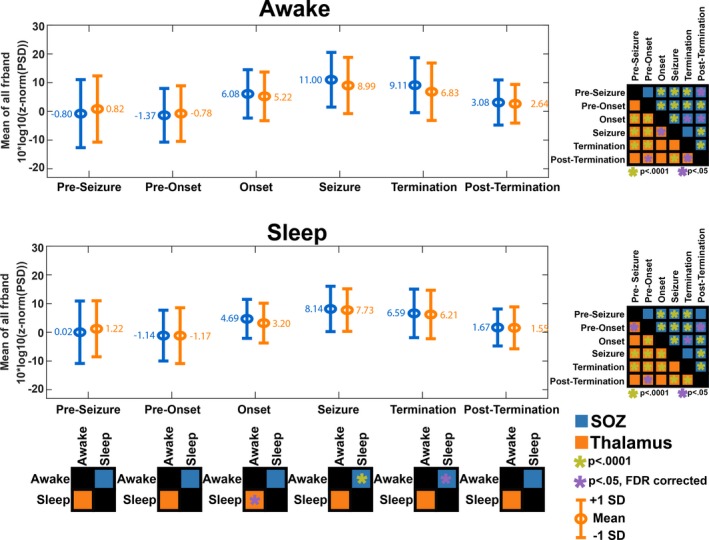
(A and B) PSD of SOZ and ANT were independently assessed for wakefulness and sleep. (C) Highlights the comparison of the PSD of ANT for seizures that occur when awake compared to seizures that occur during sleep, across the different stages of seizures. A similar comparison was made for SOZ. (D and E) Post Hoc comparison of PSD changes across seizure stages in ANT and SOZ

The thalamic PSD was higher at onset for seizures that occurred when awake compared to seizures that occurred during sleep (*t* = 2.83, pFDR = 0.005) (Fig. 6C). For example, the PSD in the delta band was higher for seizures that had onset during wakefulness compared to those that occurred during sleep (*t* = 2.95, pFDR = 0.005) (Fig. 4B). Furthermore, the thalamic PSD was found to increase from baseline through onset into a midseizure and decrease toward termination (Fig. 6D and 6). However, when comparing PSD between the thalamus and SOZ, there was no difference across all stages of both awake and sleep seizures (Fig. 6A and 6).

## Discussion

We herein report results from the first human study that compares peri‐ictal changes in power spectra of recorded LFP with clinical macroelectrodes from anterior thalamus and epileptogenic cortex in mesial temporal lobe epilepsy. Previous studies investigated the spectral dynamics between ANT and hippocampus in a preclinical model of TLE.^26^, ^27^ The motivation for studying the ANT stems from preclinical studies that demonstrated its ability to regulate seizure onset and propagation in mesial temporal lobe seizure,^9^, ^10^, ^28^, ^29^ thereby potentially making ANT a clinically relevant target in TLE.^11^ Furthermore, the ANT is integral to the limbic circuit that regulates neurobehavioral functions, including memory, mood, and emotions.^17^, ^30^, ^31^ It has been shown that open‐loop deep brain stimulation (DBS) of ANT can disrupt these physiological processes and worsen sleep,^32^ depression, and memory.^11^, ^33^ To improve the tolerance and effectiveness of ANT DBS, the research impetuous is to identify biosignatures for targeted perturbation with high temporal precision. In this context, LFP fluctuations provide a surrogate for neuronal activity, can be detected with high fidelity and temporal precision, and provide mechanistic insights about the information flow across neural networks.^34^ The pertinent findings in the study are: 1. the power (squared amplitude of the oscillations) of thalamic field potentials at baseline was significantly lower than the cortex, 2. temporal changes of ictal power of neural activity within ANT mimics that of the SOZ, is increased significantly at seizure onset and is distinct for FIAS and FBTCS and 3. SOV affects changes in the spectral content of LFP within thalamus at seizure *onset*.

Generated by postsynaptic dendritic activity, the LFP reflects the summed synaptic currents elicited by one neuronal population on another.^35^ The amplitude of LFP is determined by the structural organization and functional interaction between the generators.^35^ The heterogeneous and scattered arrangement of cells within the three subnuclei of ANT can yield lower amplitudes than those expected in the orderly arranged neuronal clusters within the hippocampus.^36^ Stereotyped high‐frequency (250–300 Hz) bursting and tonic neuronal activity during wakefulness and sleep were recorded from the ANT in preclinical model.^37^

Studies have demonstrated that the generation and propagation of focal seizures require coordination within a spatially distributed network.^38^ Although the precise pathways of seizure propagation may vary within and across patients, the role of subcortical relay structures like the thalamus in the generalization of seizures has been emphasized.^27^, ^39^ The ANT is synaptically interconnected to the mesial temporal regions. In a preclinical study, neuronal firing rate in ANT was increased with the onset and progression of hippocampal seizures.^28^ In our study, using increased broadband power spectra of LFP as a surrogate for local cortical activation,^40^ we confirmed the progressive increase in thalamic population activity with the onset and progression of mesial temporal lobe seizures. The findings were consistent across all seizure types although the neuronal population responses within ANT were highest during the seizure for FBTCS. Changes in thalamic field potentials have been recorded in the past with referential electrodes and, therefore, could be secondary to volume conduction of synchronous cortical activity.^41^ However, our analysis of thalamic field potentials was performed in the bipolar montage that overcomes volume conductance effects and reflects locally generated neuronal activity.^42^

This study also provides evidence that clinical seizure subtypes can be predicted at seizure onset by studying the neural activity within and outside the SOZ.^43^, ^44^ This evidence is in contrast to conventional teaching that seizure propagation and not onset determines seizure subtypes. The statistically significant difference in the distribution of frequency‐specific power spectra between SOZ and ANT at the onset of FIAS and FBTCS seizures supports this hypothesis, that is, that the neural dynamics at seizure onset can vary with seizure subtypes.^45^ Further studies are needed to confirm the potential of thalamocortical network dynamics to determine seizure subtypes from their onset. The increased neural activity in gamma, ripple, and fast ripple bands within the SOZ at the seizure onset and progression is consistent with previous studies.^25^, ^46^

The thalamus is a potential “choke point” in focal epilepsy.^47^ The ability of the ANT to modulate the limbic network is demonstrated by improved seizure outcome in open‐loop DBS of the thalamus studies, which possibly also modulates intrahippocampal synchrony.^48^ Targeted intervention preictally may be advantageous as it may not only prevent the seizure altogether but may also circumvent untoward consequences (e.g., depression) of frequent open‐loop ANT stimulation. The early changes in the power spectra in the ANT and SOZ before seizure onset (i.e., our *pre‐seizure* and *pre‐onset* stages) adds to the growing body of evidence that seizures are preceded by discrete dynamic changes at the neuronal and network levels that extend beyond the seizure “focus.”^25^, ^46^ Since LFPs reflect inputs from cortical or subcortical areas in addition to local network interactions,^34^, ^49^ the changes in LFP power spectra preceding seizure onset may reflect diverse neural states (e.g., synchrony, changes in local excitation and inhibition)^50^ involving the ANT and SOZ that could be targeted to disrupt early ictal organization of seizures.

The spectral content of the thalamic LFP was found to be different for seizures that had onset during wakefulness than sleep. This state‐dependence of power spectrum on vigilance reflects a change in the temporal structure of the LFP. The decrease of power in the delta band and an increase in theta and alpha bands at seizure onset during sleep reflects slow state changes in the thalamus that may be associated with heightened vigilance or arousal. It has been shown that the activity in neural ensembles within ANT can be influenced by an abrupt transition in states of vigilance (e.g., ictal arousal from sleep).^51^ Indeed, clinical studies have demonstrated that ANT DBS can induce arousals during sleep.^32^ Also, the firing pattern (tonic vs. burst firing) of thalamic neurons varies with different SOV.^37^, ^51^ Chemical parcellation of the human ANT demonstrated *core* and *matrix* cells distributed variably between the three subdivisions of ANT nuclei.^36^ Unlike the *core* cells that have spatially distinct projections, the *matrix* cells have diffuse cortical projections that are involved in the arousal and widespread activation of the cortex.^52^

### Limitations

Antiseizure drugs (ASD) can influence neural activity, thereby confounding the interpretation of observed peri‐ictal changes in the LFP. However, our study was focused on comparing changes in neural activity between ANT and SOZ. Taking in consideration that current ASDs have a heterogeneous effect on the brain, it is unlikely that the employed population statistics were biased by the preferential influence of ASD in one brain region over others (e.g., SOZ or ANT). As our study was focused on comparing power spectral between ANT and SOZ, we have limited our analysis to only one pair of electrodes in the SOZ. There are no standardized parameters to delineate SOZ, and hence, we have adopted a well‐published quantitative method (EI) and then selected the channel that showed the earliest ictal changes using line length. A future study should incorporate all the recorded electrodes or at a minimum the electrodes within SOZ to map the circuit dynamics essential for ictal propagation from mesial temporal lobe seizures to the ANT. A major challenge in neuroscience is the interpretation of LFP changes. Establishing the relationship between synaptic inputs reflected in LFPs and spiking activities that represent neuronal outputs is not straightforward. The task is even more daunting when comparing brain regions like the thalamus, where spiking activity is less correlated, and the cortex, where spiking is more correlated due to the ordered arrangement of current sources and sinks. Due to ethical constraints, recording peri‐ictal neuronal microactivity from the thalamus in patients with epilepsy may not be feasible at present. Although the concurrent recording of LFPs and unit activities can be performed with commercially available hybrid macro–micro electrodes, these implantations during SEEG were limited to structures that are amenable to surgical resection. As thalamus is not resected in epilepsy surgery, implantation of penetrating microwires in the thalamus outside the intraoperative recordings may not be justified ethically. We expect that neural modeling would bridge the gap in understanding the involved macroscale dynamics.^53^

## Conclusion

Analyzing LFP signals recorded by sEEG macro electrodes from the thalamus and cortex, we compared the peri‐ictal changes in power spectra within the ANT and SOZ in mesial temporal lobe epilepsy. We demonstrated a significant increase in ictal neural activity within the ANT that appeared similar to ictal changes within the SOZ, thereby providing direct supporting evidence that focal seizures can recruit distant subcortical structures at seizure onset. Our findings confirm that widespread changes in neural dynamics extend beyond the seizure focus and that ictal activity can be detected within the ANT based on spectral analysis, thus presenting an opportunity for the development of a biomarker for closed‐loop ANT DBS for TLE. Also, our analysis yielded differences in spectral signatures in ANT compared to SOZ during seizure onset for different seizure subtypes, thus potentially facilitating seizure subtype classification earlier on in seizure evolution. Finally, we showed that state of vigilance affects the spectral content within the thalamus at seizure onset shedding more light on the role of the thalamus and the state of vigilance in the evolution of the epileptic brain toward seizures.

## Author Contributions

Sandipan Pati MD from the University of Alabama at Birminghamcontributed to design and conceptualized the study; acquisition of data; interpretation of the data, and drafted the manuscript for intellectual content. Diana Pizarro BSSE from theUniversity of Alabama at Birmingham analyzed the data and revised the manuscript for intellectual content. Adeel Ilyas, MD from the University of Alabama at Birmingham contributed to the interpretation of data and drafted the manuscript for intellectual content. Ganne Chaitanya, MBBS; PhD from the University of Alabama at Birmingham designed the study; analyzed and interpreted data, and revised the manuscript for intellectual content. Emilia Toth, PhD from the University of Alabama at Birmingham analyzed the data and revised the manuscript for intellectual content. Auriana Iranejaad from the University of Alabama at Birmingham played a major role in the acquisition of data. Kristen Riley, MD from the University of Alabama at Birmingham contributed to implant strategy, acquisition of data, and revised the manuscript for intellectual content. Andrew Romeo, MD from the University of Alabama at Birmingham contributed to implant strategy, acquisition of data, and revised the manuscript for intellectual content. Leon Iasemidis,PhD from the Louisiana Tech University, Ruston, LA revised the manuscript for intellectual content.

## Conflict of interest

None.

## Supporting information


**Table S1.** Demographics of the patient with details of seizure semiology, surgical workup, and treatment outcome
