# Supplementary material for: Spectral organization of focal seizures within the thalamotemporal network
Source: Ann Clin Transl Neurol. 2019 Aug 30;6(9):1836–48. doi: 10.1002/acn3.50880 (PMC6764631; doi:10.1002/acn3.50880)
Supplement: Supplementary file 1 — Table S1. Demographics of the patient with details of seizure semiology, surgical workup, and treatment outcome [file ACN3-6-1836-s001.docx]

| **Patient index** | **Age at sEEG (y), Gender** | **Epilepsy onset (y)** | **Epilepsy Duration (y)** | **Seizure semiology** | **MRI** | **ANT implant side** | **Electrodes implanted** | **Seizures studied** | **sEEG SOZ** | **SOZ channels studied** | **Surgical treatment** | **Histopathology** | **Last follow up & outcome** |
| --- | --- | --- | --- | --- | --- | --- | --- | --- | --- | --- | --- | --- | --- |
|  |  |  |  |  |  |  |  |  |  |  |  |  |  |
| 1 | 26,M | 8 | 18 | FIAS (arousal→manual automatism) | No lesion | L | 12-L, 8-R | ES=1, FIAS=3 | L MTLE plus (orbF) | L Amy | L OF resection Planned for RNS LH | gliosis | Engel I at 16 mo |
|  |  |  |  |  |  |  |  |  |  |  |  |  |  |
|  |  |  |  |  |  |  |  |  |  |  |  |  |  |
| 2 | 33,M | 1 | 32 | FIAS (oro-manual automatism→dystonic arm posture) FBTCS (arousal→head version→tonic clonic) | R postTO encephalomalacia | R | 20-R | FIAS=2, FBTCS=2 | Multifocal (R MTL, Ins, orbF) | R A Ins | Palliative R ATL extended to STG, A Ins | FCD type II | Engel IV at 9 mo |
|  |  |  |  |  |  |  |  |  |  |  |  |  |  |
| 3 | 30,F | 12 | 18 | FIAS (arousal→stare, confusion) FBTCS (arousal→vocalization, head version →tonic clonic) | No lesion | L | 9-L | FIAS=4, FBTCS=3 | L MTLE (Lat STG) | LH | Declined resection. Planned RNS LH | na | na |
|  |  |  |  |  |  |  |  |  |  |  |  |  |  |
| 4 | 41,F | 38 | 3 | FIAS (experiential aura→manual automatism) FBTCS (experiential aura→head version and tonic clonic) | L Amy+H increased signal on FLAIR | L | 9-L | FIAS=2, FBTCS=3 | L MTLE | L Amy | Patient deferred L ATL | na | na |
|  |  |  |  |  |  |  |  |  |  |  |  |  |  |
|  |  |  |  |  |  |  |  |  |  |  |  |  |  |
| 5 | 50,F | 40 | 10 | FAS (abnormal taste→paresthesia) FIAS (lip smacking→behavioral arrest) FBTCS (abnormal taste→arm dystonic→ tonic clonic) | No lesion | R | 12-R | ES=1, FAS=3 , FIAS=1 | R MTLE plus (Ins-Oper) | RH | R ATL extended to STG and A Ins | Neuronal loss FCD type I | Engel I at 9 mo |
|  |  |  |  |  |  |  |  |  |  |  |  |  |  |
| 6 | 57,F | 52 | 5 | FAS ('spaced out feeling'→warmth sensation) FIAS (loss of awareness→manual automatism) | No lesion | R | 13-R | ES=2, FAS=2, FIAS=2 | R MTLE -Tp | RTp | R ATL including Tp | Gliosis | Engel I at 12 mo |
|  |  |  |  |  |  |  |  |  |  |  |  |  |  |
| 7 | 58,F | 16 | 42 | FIAS (gustatory aura, excessive salivation→facial clonic activity) | R HS, R P encephalomalacia | R | 14-R | E=2, FIAS=3 | Multifocal (R MTL, R P) | R Amy | palliative LITT RH+R Amy | na | Engel II at 5 mo |
|  |  |  |  |  |  |  |  |  |  |  |  |  |  |
|  |  |  |  |  |  |  |  |  |  |  |  |  |  |
| 8 | 61,M | 40 | 21 | FIAS (light headedness→chest tightness, neck pain→confusion) | R HS, L H malrotation | R | 13-R, 7-L | E=2, FIAS=2 | R MTLE | RH | R ATL | HS | Engel I at 12 mo |
|  |  |  |  |  |  |  |  |  |  |  |  |  |  |
|  |  |  |  |  |  |  |  |  |  |  |  |  |  |
| 9 | 48,F | 41 | 7 | FAS (auditory hallucination→sense of body vibrating) FBTCS (oro-manual automatism→tonic clonic) | No lesion | R | 14-R | FAS=4 | R MTLE (Lat STG) | R STG | RATL extended to STG | Gliosis | Engel I at 6 mo |
|  |  |  |  |  |  |  |  |  |  |  |  |  |  |
|  |  |  |  |  |  |  |  |  |  |  |  |  |  |
| 10 | 24,M | 1 | 23 | FIAS (loss of awareness→behavioral arrest) FBTCS (arousal→ automatism →tonic clonic) | L HS | R | 12-R, L-8 | FIAS=1, FBTCS=3 | Bitemporal (R MTL-Tp, L MTL) | R Amy | R Tp resected, RH spared, planned B/L H RNS | FCD type I | Engel II at 6 mo |
|  |  |  |  |  |  |  |  |  |  |  |  |  |  |
|  |  |  |  |  |  |  |  |  |  |  |  |  |  |
| 11 | 29,M | 11 | 18 | FIAS ('freeze' or behavioral arrest→oral automatism→curls neck, body around midline axis 'cursive' movement) | L H+L medO atrophy | L | 16-L | FIAS=1, FBTCS=4 | LH EEG onset preceding clinical changes but semiology not consistent with anatomy. Consensus was non localized SOZ | L Amy | Offered ANT DBS | na | na |
|  |  |  |  |  |  |  |  |  |  |  |  |  |  |
|  |  |  |  |  |  |  |  |  |  |  |  |  |  |
| y-years, M-male, F-female, ES-electrographic seizure, FAS-focal aware seziures, FIAS-focal impaired awareness seizures, FBTCS-focal to bilateral tonic clonic seizures, MRI-magnetic resonance encephalography, R-right, L-left, B/L-bilateral, T-temporal, P-parietal, F-frontal,O-occipital, MTLE-mesial temporal lobe epilepsy, orb-orbito, Ins-insula, Oper-opercular, Amy-amygdala, H-hippocampus, S-superior, Lat-lateral, A-anterior, G-gyrus, Tp-temporopolar, ATL-anterior temporal lobectomy, RNS-responsive neurostimulaiton, LITT- Laser Interstitial Thermal Therapy, DBS-deep brain stimulation, FCD-focal cortical dysplasia | | | | | | | | | | | | | |
